# Exploring Care Needs of Partners of Transgender and Gender Diverse Individuals in Co-Transition: A Qualitative Interview Study

**DOI:** 10.3390/healthcare11111535

**Published:** 2023-05-24

**Authors:** Isabeau Van Acker, Alexis Dewaele, Els Elaut, Kariann Baetens

**Affiliations:** 1 Faculty of Psychology and Educational Sciences, Department of Experimental Clinical and Health Psychology, Ghent University, Henri Dunantlaan 2, 9000 Ghent, Belgium; 2 Centre of Sexology and Gender, Ghent University Hospital, Corneel Heymanslaan 10, 9000 Ghent, Belgium

**Keywords:** care needs, gender diversity, gender-affirming transition, intimate partner, intimate relationship, professional support, transgender

## Abstract

Scientific knowledge on the impact of a gender-affirming transition on intimate partners of transgender and gender diverse (TGD) individuals is limited. It is unclear which care needs partners have and which role health care professionals can play during this transition process. The aim of this study was to explore the unique experiences and care needs of people partnering with TGD people in the context of a gender-affirming transition. A qualitative research method was chosen, and a semi-structured interview was conducted with a sample of nine participants. After transcription, thematic analysis was used to analyse the data. Three main themes, with three subthemes each, were identified: (1) intrapersonal processes, with (1a) the process of acceptance, (1b) concerns surrounding the medical transition and (1c) impact on sexual orientation as subthemes; (2) dyadic processes, with (2a) the importance of mutual commitment, (2b) experiences regarding intimacy and (2c) relational growth as subthemes; and (3) perception of support, with (3a) need for support, (3b) the importance of support and (3c) evaluation of support as subthemes. The results suggest that health care providers can help partners to navigate the process of a gender-affirming transition; however, the care needs of partners are currently not satisfied with the available professional support.

## 1. Introduction

In recent years, the social visibility of transgender and gender diverse (TGD) individuals in society has increased significantly in the Global North [1,2,3,4]. TGD refers to all forms of gender variance in individuals and is therefore broader than the binary gender model [5]. TGD individuals are defined as anyone whose gender identity differs from their assigned gender at birth, which is typically determined by sex [6]. Individuals who experience a consistency between their assigned and experienced gender identity are described as cisgender. The growing social awareness of TGD individuals in the Global North has been accompanied by a rise in research on this population [7]. However, it is noteworthy that most of the literature revolves around TGD individuals themselves. The experiences of those in the social context of TGD individuals, such as their intimate partners, are much less addressed in research [1,2,8,9]. Although there has been recent steady growth in the literature on partners of TGD individuals [8,9,10,11], gaps in current knowledge remain and there is a need for further research. 

A brief review of key literature on intimate partnerships in the context of a gender-affirming transition is presented below, followed by a discussion on the professional support available to partners of TGD people in the Belgian context. Finally, the aims and research questions of the current study are described.

### 1.1. Co-Transition

TGD individuals may choose to change their characteristics to better align with their experienced gender identity [1], also referred to as a “gender-affirming transition” or “transitioning”. A further distinction can be made between a medical transition and a social transition [1,8]. A medical transition refers to gender-affirming medical interventions (GAMI) that help to form gender-congruent physical characteristics [1]. A social transition involves the TGD person making gender-affirming changes at a social level. Examples include changes in gender expression (e.g., through clothing and appearance), as well as communicating with the environment about gender identity [1,12]. A gender-affirming transition is highly individualised and unique, as it is shaped by the needs and wishes of each individual TGD person. However, transitioning also possibly has effects on people in the TGD person’s environment. To study only the intra-individual processes would therefore fail to capture reality. It is important not to lose sight of the interpersonal dynamics, including the intimate relationship [13].

According to Bradbury and Karney (2019), an intimate relationship is distinguished from other forms of social relationships, based on four criteria: it involves bidirectional interdependence, is considered to be personal, close, and is or has the potential to be sexual [14] (pp. 22–25). In intimate relationships, gender identity and expression play an important role in several domains. For instance, it affects physical attraction, partner choice, and intimacy [8]. If a partner comes out as TGD and initiates a gender-affirming transition, this may affect the other partner(s) and their interpersonal relationship [13,15]. Theron and Collier (2013) coined the term “co-transitioning” to refer to the changes that the partner(s) and the relationship undergo during a gender-affirming transition [15]. The scholars argue that the relationship, as well as the partner(s), are transitioning at the same time as the TGD person. 

A few studies provide data on the number of partner relationships among TGD people. A large-scale study (*N* = 1778) on TGD individuals within the EU concludes that the majority of participants did not have a partner or other relationship at the time of the study [4]. Of the participating TGD people, 29% were living with their partner(s) and 24% were in a relationship without living together. Similar results can be found within Belgium. For instance, in the study of Burgwal et al. (2023) (*N* = 936) on experiences of violence of TGD people, it was found that 50.3% of respondents were in a partner relationship at the time of the study [16]. Furthermore, it appeared that the majority of partners identified as women [4]. Although these figures cannot be generalised, they reflect the fact that a part of the TGD population in the EU and in Belgium is in an intimate relationship. What these results do not mention, however, is when the intimate relationships were initiated and whether they remained in the context of a social and/or medical transition. Scheim and Bauer (2015) found a difference in the timing of trans men’s and trans women’s gender-affirming transitions. They argue that trans men often transition earlier in life than trans women, which means that trans women are often already involved in a long-term partner relationship before they inform their partner of their gender identity [17].

It is common among people with a TGD partner to experience doubts about their sexual orientation, which can be defined using three dimensions: (1) how a person labels themselves (i.e., self-identification), (2) the person’s sexual partners (i.e., sexual behaviour), (3) and who a person feels attracted to (i.e., sexual attraction) [4,18]. Furthermore, concerns about the survival of the relationship once transitioning is initiated was also found in previous research, as well as feelings of anger, sadness, grief and confusion once the gender identity and wish to transition were disclosed [15]. However, contrary to earlier beliefs [19,20,21], more recent studies show that a healthy, satisfying relationship with TGD people is possible and that an intimate relationship can endure a gender-affirming transition [1,11,15,22,23]. During the transition process, a process of acceptance can be described for both family members and partners of TGD people [24,25]. Several authors have reflected on its potential course, the most prominent being the models of Lev (2004) and Emerson and Rosenfeld (1996) [24,25]. Based on her clinical experience, Lev (2004) defines four stages in the process of acceptance by partners and family members, which she refers to as the “Family Emergence Stages”: (1) discovery and disclosure, (2) turmoil, (3) negotiation and (4) finding balance [24]. In contrast to Lev, Emerson and Rosenfeld (1996) distinguish five stages: (1) denial, (2) anger, (3) bargaining, (4) depression and (5) acceptance [25]. Emerson and Rosenfeld’s model emphasises that the stages are not linear, that individuals do not necessarily experience all of them, and that each stage may be experienced uniquely by individual family members [25].

The limited research further indicates that transitioning can have a strong impact on several areas of an intimate relationship, particularly in terms of sexual intimacy [1]. As intimacy can be understood as “partners’ general sense of closeness” (p. 276) [26], sexual intimacy is defined as sexual behaviour in combination with a general sense of closeness. The fact that TGD people can initiate a gender-affirming transition at any time in their lives creates a wide variety of situations in relationships. The few studies that have been conducted among partnerships show that considerations about sexual intimacy occur mainly among partners coupled before the transitioning [8]. Transitioning partnerships go through a process of reshaping their sexual intimacy, including exploring the changing body and changing sexual roles. During this development, sexual scripts can change and take on a new meaning [1]. The physical changes brought about by a medical transition can reduce negative feelings due to gender dysphoria in the TGD partner, which in turn can lead to new sexual experiences and increased sexual intimacy within the partnership [8]. However, these physical changes can at the same time cause reluctance and discomfort in and between partners [2,8]. In contrast to sexual intimacy, little is known about physical intimacy (i.e., physical proximity in combination with a general sense of closeness, which can include sexual behaviour) in partner relationships that include at least one TGD person.

In addition to this intra-individual and dyadic impact, a TGD individual and their partner(s) are embedded in a broader sociocultural context. A partnership’s social environment may respond to gender diversity and transitioning in varying ways. Because of gender identity and expression, TGD individuals experience high levels of discrimination, prejudice and aggression [2,16,27]. According to Meyer’s (2003) minority stress model, the stigmatisation and discrimination of people belonging to social minority groups leads to high levels of stress, “minority stress”, which in turn has a potential negative impact on mental health [28,29,30,31]. In the context of the minority stress model, it can be argued that partners of people from minority groups may also feel or experience the effects of these minority stressors themselves. This is captured in the dyadic stress theory, which states that stress experienced by partners, or the transmission of stress from one partner to another, can have a negative impact on all partners [32,33]. The stigma associated with the intimate relationships of people with TGD is an example of such a stressor and can affect the mental well-being of all partners and the relationship quality [28,29]. So, even when partners do not themselves belong to a gender minority, available evidence suggests that the minority stressors present can have an effect on the partner(s) as well [8].

### 1.2. Professional Care for Partners

Little is known about how partners cope with these challenges and the specific care needs they experience. Because of the lack of research, there is also limited information about professional care and support for partners of TGD people [24], despite evidence showing that being in a supportive partnership can be an important protective factor for people facing stressful life situations [34]. The Standards of Care for the Health of Transgender and Gender Diverse People, version 8 (SOC-8), do not provide specific guidelines, but mention that “the inclusion of sexual and/or romantic partners in transition-related health care can facilitate the process of “co-transitioning” and can also support sexual growth and adjustment both in the individual as well as in the relationship.” [35] (pp. 165–166). Some scholars and clinicians do, however, provide recommendations and guidelines on providing psychotherapy for TGD partnerships and partners of TGD individuals [24,36,37,38,39,40]. More research on the experiences and care needs of partners can contribute not only to knowledge, but also to better professional care so that TGD people and their partners can be better supported.

In Belgium, partner support is limited. There are a few online forums and support groups where partners can get in touch with peers. In terms of professional initiatives, people can visit the Transgender Infopoint, a support organisation and knowledge centre based at the UZ Gent, were everyone can ask questions on gender diversity and transgender issues [41]. Furthermore, the Transgender Infopoint has set up a support group for (former) partners of TGD people, which meets throughout the year in several locations in Flanders. This organisation also organises one-on one contact between (ex-)partners [41]. There are currently no specific initiatives in the field of professional mental health care. Partners can, of course, always enter psychotherapy, although it is unclear to what extent the therapist will be familiar with TGD issues.

### 1.3. Current Study

Many scientific questions remain about partners of TGD individuals, whose perspectives are still underrepresented in research. There are a handful of studies that have focused on this target group and some findings have emerged, but there is a need for further exploration and replication. The current study aims to contribute to the existing body of knowledge about partners of TGD people, based on their unique experiences. Using the information gathered in the literature review, the following research questions were formulated: (1) “How do partners of TGD people experience their partner’s gender-affirming transition?” and (2) “How do partners of TGD individuals experience informal and professional support in relation to their partner’s gender-affirming transition?”.

## 2. Methods

A qualitative research method and thematic analysis design was chosen to address the research questions. Qualitative research allows us to gain an in-depth understanding of individuals’ perspectives on a particular phenomenon [42]. As a result, the primary focus of this study was on the experiences and needs of the partners of TGD individuals. Given the limited research on the experiences and care needs of individuals with TGD partners, it was deemed important to explore this topic in depth as an initial step. The Standards for Reporting Qualitative Research (SRQR) were followed in the reporting of the present study (see Appendix A) [43].

### 2.1. Sample

The research project was approved by the Medical Ethics Committee of Ghent University Hospital before it was initiated (registration number B670201941011). A total of nine partners of TGD individuals participated. This is in line with the guidelines of Smith (2015), who recommends recruiting six to fifteen participants in this type of research [42]. Due to the challenging nature of reaching the target population, the decision was made to use convenience sampling via social media. This involved distributing a call with a digital flyer via the Transgender Infopoint Facebook page. The inclusion criteria for participants were the following: (1) the participant is at least 18 years old, (2) the participant has been in an intimate relationship for at least two years with a TGD individual, and (3) the relationship existed before or began during the TGD partner’s gender-affirming transition. The minimum relationship duration of two years was assessed as short enough to recruit a significant proportion of the target population, yet long enough for participants to report relevant experiences of being together with a TGD person in transition. Only partners of individuals that had received GAMI were recruited in order to best capture the impact of the transition process. The conceptualisation of an intimate relationship was determined by how the participants themselves defined it. No other inclusion criteria were used to allow the diversity of this population to be reflected in the findings and to provide as complete a picture as possible of the unique experiences of TGD people’s partners.

Ultimately, nine partners participated in this study (see Table 1). The age of the participants ranged from 21 to 52 years old. One participant reported having a Portuguese nationality, while the remaining participants were of Belgian nationality (*N* = 8). Despite attempts to recruit partners who did not identify as cisgender women, eight participants identified as cisgender women. One participant identified as non-binary. In terms of sexual orientation, the most common identity was ‘pansexual’ (*N* = 4). Other sexual orientation identities that were mentioned were: lesbian (*N* = 2), bisexual (*N* = 1) and heterosexual (*N* = 1). One participant said she did not use a particular term for her sexual orientation and described herself as “not 100% heterosexual”. The participants’ TGD partners identified as transgender men (*N* = 3), men (*N* = 1), transgender women (*N* = 1), women (*N* = 3) and non-binary transgender men (*N* = 1). Relationship duration ranged from 4 to 27 years, with a median relationship duration of 9 years. Two participants were aware of their TGD partner’s gender identity prior to the start of the relationship, and one participant was aware of her partner’s cross-dressing. To protect the privacy of the participants, pseudonyms were used in the reporting of the results. 

### 2.2. Materials

An in-depth semi-structured interview was used to explore the experiences and care needs of people with TGD partners (see Appendix B). This was chosen because a semi-structured interview allows for flexibility and in-depth answers that can be elaborated on if necessary. With the permission of the developers, the interview guide was partly based on “The Transgender Partner Experience Interview” by Platt and Bolland (2018) [8]. Relevant questions in the Transgender Partner Experience Interview were translated to Dutch and, where necessary, slightly modified to facilitate correct interpretation. The remaining questions were formulated according to the current research questions. The interview guide was piloted in one pilot interview with a partner of a TGD person, but no substantial changes were made based on feedback. The final topic list consisted of questions about the participants’ demographics, their TGD partner’s coming out, the history and future of their relationship, the reactions of people in their environment, and their needs and experiences with support.

### 2.3. Procedure

Participants were recruited through two public social media posts. Of those who responded to the call, it was first verified that they met the inclusion criteria. They were then informed again about the purpose of the study and how their data would be processed. A total of nine in-depth interviews were conducted. The interviews took place in a quiet room in the participants’ homes and were always conducted by the same researcher, Van Acker I. An informed consent form was read and signed by all participants beforehand. With participants’ permission, a digital audio recording of the interviews was made. No compensation was given to participants; participation in this study was entirely voluntary.

### 2.4. Data Analysis

After data collection, the interviews were transcribed from the digital audio recordings. Any information from which participants could be identified was immediately pseudonymised. The data were analysed using inductive thematic analysis by one researcher, Van Acker I. This qualitative method allowed for the flexible identification of recurring themes within the interviews [42,44]. It was chosen because it focuses on the content of what is said, rather than how it is said [45]. This makes thematic analysis highly appropriate for research questions related to perspectives and experiences, as is the case in this study. This study took an inductive or data-driven approach: the analysis was grounded in the data, rather than from predetermined coding schemes, theories or concepts [44]. The themes were identified based on common findings and patterns within the participants’ narratives. The Nvivo 12 software was used to analyse the data digitally [46].

Thematic analysis typically involves six steps [42,43,44], which were also carried out in this study:

1. The interviews were reviewed twice after transcription in order to become familiar with the data. The transcription itself also increased the understanding of the content of the interviews.
2. The data were coded, with the initial codes matching the raw data as closely as possible. In total, approximately four coding rounds were conducted. Throughout this process, codes were expanded, split into multiple codes or merged together. The relevance of the codes was based on the formulated research questions.
3. Recurring or similar codes across the interviews were grouped into coherent and meaningful themes, underpinned by a key analytic point. The possible relationships between these themes were analysed, creating a distinction between the main themes and subthemes.
4. The initial themes were analysed once more and refined. Efforts were made to achieve good internal homogeneity and external heterogeneity. The aim was to make the data within themes sufficiently coherent and the data from different themes clearly distinguishable.
5. The themes were defined and named. A graphical overview of the relationships between the themes found was created in the form of a thematic map.
6. The themes found were selected and reported. This can be found in the Section 3.

### 2.5. Quality Control

A researcher has an active role in the collection and analysis of data, making it impossible to eliminate researcher subjectivity from study findings [42]. In this study, data were collected and analysed by one researcher, Van Acker I. Reflection on her positionality is necessary. The characteristics of the researcher that potentially had an impact involve her being a young, white, Belgian, queer, cisgender woman, a master’s student in psychology, coming from a middle-class family and being a newcomer to the research field. Prior to the data collection, due to information given in the online call for participants and during the first contact through mail, participants had knowledge on the position of the researcher as a psychology student. During data collection, being a white, Dutch-speaking person with a feminine gender expression may have made participants, almost all of whom identified as white, Belgian and female, feel more comfortable sharing their experiences with the researcher. The researcher identifying as queer was sometimes disclosed at the start of the interview when participants were welcomed to ask questions about the study or researcher. This knowledge may have made it easier to establish a trusting relationship with non-heterosexual participants. In contrast, this characteristic may have made other participants wary of expressing critical ideas about their TGD partner and the gender-affirming transition. For young participants, the young age and student status of the researcher may have made them feel more at ease, in contrast to older participants who had already graduated. In data analysis, this position as a newcomer to the research field may have led to a lack of knowledge and nuance. However, this may have also had the benefit of the researcher looking at the data with a fresh perspective. Finally, a more positive interpretation of some of the participants’ feelings may have stemmed from her own identity as a queer person.

Several steps were taken to improve the quality of data collection and analysis. This was based on the work of Guba and Lincoln, who developed two criteria for assessing the quality of qualitative research: (1) trustworthiness and (2) authenticity [47,48,49,50]. In order to achieve the highest possible quality, three methods were used in the current study. Firstly, member checking was used. Participants were asked to evaluate the transcribed data of the interview in which they participated for correctness and the extent to which the data corresponded to their perspectives. Secondly, a document trail was created. In this document trail, the researcher documented decisions and insights during the research process. Finally, the method of reflexivity was used to ensure reliability and authenticity. Here, the principal researcher provided a description of personal social and cultural embeddedness and how this potentially influenced the research process, which can be found above.

## 3. Results

**Figure 1:**
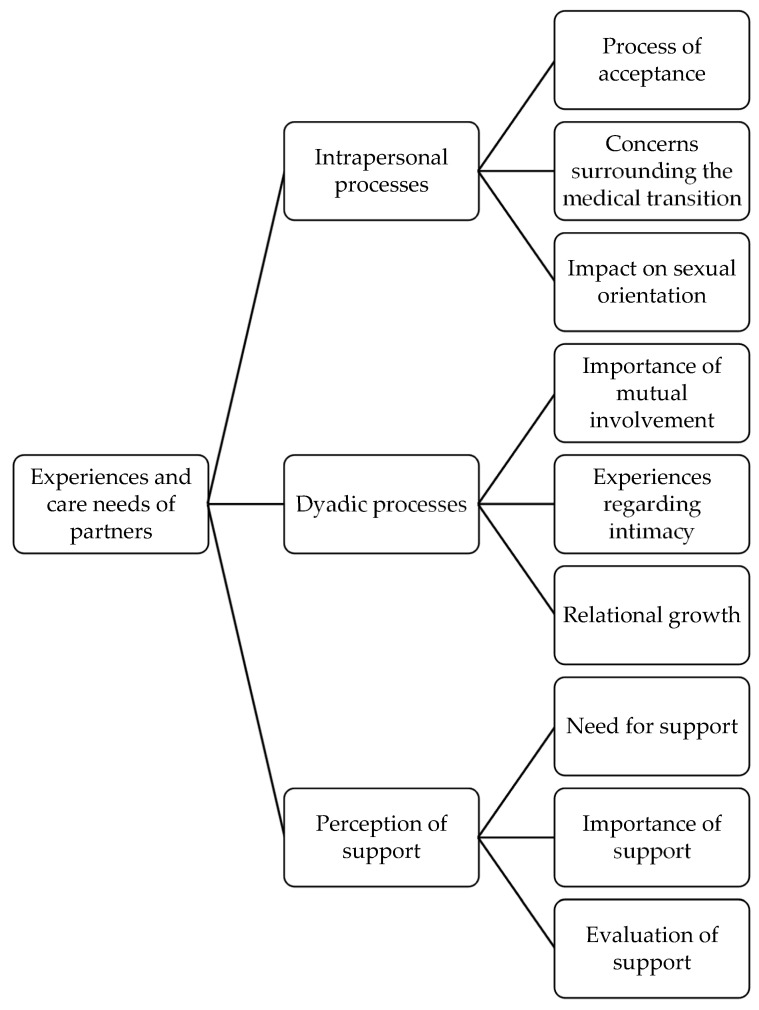
Thematic map of the themes.

Based on the research questions, three themes were identified during the analysis: (1) intrapersonal processes, (2) dyadic processes and (3) perception of support. The first theme, intrapersonal processes, describes participants’ perceptions of experiencing internal processes during their partner’s transitioning and its impact on them. Three sub-themes were identified, namely (1a) the process of acceptance, (1b) concerns about medical transition and (1c) impact on sexual orientation. The experience that a gender-affirming transition is a process involving both partners and that there are changes within the intimate relationship is covered by the second theme, dyadic processes. A further distinction was made between the sub-themes (2a) the importance of mutual commitment, (2b) experiences regarding intimacy and (2c) relational growth. The final theme, perception of support, reflects the participating partners’ perceptions and experiences of professional and informal support in the context of a gender-affirming transition. Again, three sub-themes were identified: (3a) need for support, (3b) importance of support and (3c) evaluation of support. A graphical representation of the themes and their sub-themes can be found in Figure 1.

### 3.1. Intrapersonal Processes

#### 3.1.1. Process of Acceptance

The participants’ perspectives reflected that each partner went through individual processes after the coming out and during their partner’s gender-affirming transition. Parallel to their partner’s transition, all interviewed partners experienced a change in their feelings. The development of this process and the feelings experienced differed significantly between participants. Nevertheless, the result for all participants was the same: reconciliation with their partner’s gender identity and transition.

Regarding their TGD partner’s coming out, four participants reported having little difficulty with this. For two of these partners, the coming out took place before the start of the relationship. A third partner was already aware of her partner’s cross-dressing before they got together. Thus, these three participants were at least partially aware of their partner’s gender identity at the beginning of the relationship. They reported having no issue with their partner’s desire to initiate a gender-affirming transition. Kathleen was aware of the desire to socially and medically transition before she got together with her current partner. She stated that they had discussed this at length and agreed to this before they initiated their relationship:

*“I knew that from the beginning. So actually, I knew for over 10 years that he wanted to make the transition one day. We talked about it before we got into a relationship. I said at the time, “You have to do what you want to do. It’s your body, if you’re not comfortable with it, you have to do it. For me you don’t have to refrain from it, I will support you””.*

*(Kathleen, 41 years old, woman, pansexual)*

The remaining five participants reported that the coming out triggered negative feelings. They reported being surprised by their partner’s feelings. Other feelings reported were confusion, disbelief, sadness, fear and panic. Four of the participants had doubts about the future of their relationship as a result of the disclosure. For three of them, this uncertainty arose because their TGD partner’s gender identity did not match their sexual orientation. Emma, for example, said that she had previously only been attracted to women and therefore thought that her relationship would not last if her partner underwent a transition:

*“It was a huge shock because I think there was still a part of me that thought, “It’ll never actually happen”, but in the end it did. I remember my first thought was that we would separate. That went through my head for a while, that I thought it wasn’t going to work”.*

*(Emma, 21 years old, woman, pansexual)*

After coming out, most participants’ feelings evolved. A wide variety of experiences were reported. Some participants indicated that their feelings evolved in a gradual, subconscious way. Through small steps and time, they were able to accommodate their feelings. For others, it was a more conscious process in which they identified specific turning points or stages. Three participants talked about how this was a mentally taxing process accompanied by many negative feelings. For two of the partners, this led to a temporary termination of the relationship. 

Lien, for example, struggled with her partner’s feelings during the first year after coming out and preferred him not to transition. She spoke of a mental barrier that she initially found difficult to overcome:

*“Things were going so well, everything felt so good, I didn’t understand what the issue was. I really tried to push it away in the beginning. […] We tried to talk a lot, but then there was a lot of crying, panic attacks, not being able to breathe,… It never came to a proper conversation. There were many attempts, but it didn’t work. I couldn’t cross that mental barrier”.*

*(Lien, 23 years old, woman, lesbian)*

Another recurring experience for several interviewed partners was having to say goodbye in some way to the person their TGD partner used to be. Participants described this as saying goodbye to, letting go of, or losing their partner as they once were. Some spoke of a grieving process they went through. In some cases, this was accompanied by conflicting feelings, for example, when participants wanted to be happy for their partner being true to themselves but actually felt sad, or when they missed their partner while being in the same room as them. Kathleen described how she realised she had to say goodbye to her ‘old partner’ when she visited him in the hospital after gender-affirming surgery:

*“That’s when I broke down for the first time, because that’s when I realised my old partner was gone. “My previous partner is gone and now it’s Kevin. I have to get used to the fact that I will never get her back.” Whereas as a partner you know you’re not actually losing your partner”.*

*(Kathleen, 41 years old, woman, pansexual)*

Ultimately, a shift towards acceptance was observed in all participants. When asked how they saw the future of their relationship, all interviewed partners indicated that they saw their future together with their TGD partner. Several participants reported being happy with the steps they had taken in the gender-affirming transition. Some reported feeling happier in the relationship now than before the transition, despite the initial negative feelings.

#### 3.1.2. Concerns Surrounding the Medical Transition

The results of this study suggest that medical transition can be an intense experience for partners of TGD people. In all but one of the interviews, there were several recurring concerns about a medical transition and its consequences. 

Five of the interviewees expressed worries about changes in their partner. These participants were concerned about physical changes, changes in personality and behaviour and other consequences of a medical transition, for example, Lien, who worried about how her partner would change physically:

*“Then I started thinking about what would change and came to the conclusion that only his eyes would stay the same. His voice will lower, he will get more body hair, his face will change. He will change in every respect. Then I thought, “But then I’ll be together with a completely different person””.*

*(Lien, 23 years old, woman, lesbian)*

Uncertainty about what medical procedures their partner would eventually undergo, and what the outcome would be, made it more difficult for some participants to cope with their feelings. Clarity about which GAMIs would be carried out and when exactly they would take place emerged as an aspect that could provide a sense of peace. When this was not the case, some participants reported that they were very anxious and that it was difficult not to get answers to their questions.

In addition, physical changes, for example, after a medical procedure, could also have an impact on the interviewed partners. Some participants talked about how difficult it was for them to be confronted with their partner’s physical changes and to realise that the changes were permanent. Genital and facial gender-affirming surgery were mentioned as challenging moments by several participants, including Eline:

*“The most difficult thing was the facial surgery. I went to drop Lotte off, and when they came to get her to go to the operating room, I walked with her to the lift. There the nurse said: “This is as far as you can go, madam.” It was at that moment that I realised that this was the last time I would see her. I hadn’t thought about it that way before, but it hit me very hard at that moment”.*

*(Eline, 41 years old, woman, not 100% heterosexual)*

Gradual changes made the impact more manageable, according to four participants. Among other things, partners mentioned during the interviews that the slow changes brought about by hormone treatment helped them to cope with these changes. In addition, some participants talked about taking the gender-affirming transition one step at a time, allowing time to adjust before introducing new gender-affirming changes, as ways of reducing distress for them.

Besides concerns about changes in their partner, five participants also expressed concerns about medical complications. Several participating partners experienced worry about medical complications and indicated that this was difficult for them. The occurrence of medical complications can also be emotionally distressing, according to the interviews. The following quote from Kathleen, talking about the complications her partner experienced during his phalloplasty, illustrates this:

*“The first time the operation had to be repeated, I really broke down at his bedside. I had to stay strong for him, but the moment I had to leave the hospital I think I cried for the first time. […] That was the first time and then there were more complications. After the fourth or fifth complication, where he had to have another surgery, I completely collapsed. I could not function anymore. I couldn’t do anything at all”.*

*(Kathleen, 41 years old, woman, pansexual)*

Finally, the decision on whether or not to freeze the gametes of the TGD partner was mentioned by two partners as a difficult moment. For these partners, it was important to have the option of having their own genetic child with their partner later in the relationship. However, when it became clear that this was not an option for the TGD partners, this was difficult for the participants to deal with. In her interview, Lien expressed how she felt:

*“But now his eggs are gone too, which means that my chances of having a child with Simon are just… gone. There’s no way I can have a child between the two of us. I find it very difficult that we can’t have genetic children of our own”.*

*(Lien, 23 years old, woman, lesbian)*

#### 3.1.3. Impact on Sexual Orientation

The interviews showed that a partner’s transition can have an impact on a person’s sexual orientation, specifically regarding the dimension of self-identification and sexual attraction. Some of the participants said that their partner’s transition raised many questions and doubts about their sexual orientation. Among other things, interviewed partners wondered whether they needed to re-evaluate their own sexual orientation and how remaining in a relationship with their partner would affect this. Kim, for example, raised several questions about this aspect of their identity after their partner came out:

*“I also had a really hard time because I started questioning myself again. Many questions ran through my head, such as “If I stay with him, does that mean something about my own sexuality? Should I revise that? For me it was all clear before, should I let that go now?” And especially: “Should I go and figure this out once more and will I land on a different sexual orientation? And what if I don’t land on something else?””.*

*(Kim, 34 years old, non-binary, lesbian)*

Following this reflection process, three partners adjusted their self-identification. In all three cases, they chose a sexual orientation label that included their partner, namely “bisexuality” or “pansexuality”. Four other participants did not change how they describe their sexual orientation, even though this did not include their partner. The reason for this was mostly that they did not feel sexually attracted to people of the same gender identity as their partner. The remaining two participants used a bisexual or pansexual orientation before the gender-affirming transition and did not feel the need to revise it. 

In terms of perceived sexual attraction to their partner, seven participants reported that they were still, or sometimes even more, sexually attracted to their TGD partner. This was the case even despite the fact that for two of them, the gender identity of their TGD partner did not match their sexual orientation label. For some interviewed partners, this was an unexpected but pleasant realisation, as in the case of Emma. Originally sexually attracted only to women, she had feared that the medical transition would prevent her from being attracted to her partner:

*“I actually find Dylan more attractive now than before, which I never expected… That’s something he was also very afraid of, but actually the truth is that I like him more every day as he becomes more like his true self. Everything falls into place now. I never expected that”.*

*(Emma, 21 years old, woman, pansexual)*

Two participants reported feeling less sexually attracted to their TGD partner as a result of the medical transition. They indicated not being attracted to their partner’s changed body. Both participants expressed sorrow about this, as they had hoped that they would still be attracted to their partner despite the physical changes.

In terms of overall sexual attraction, five participants reported little to no change in their sexual attraction to others. Three cited this as the main reason for not changing their sexual orientation label. One partner, Sarah, changed the way she describes her sexual orientation to include her partner, even though she still felt primarily attracted to men:

*“I’m still mainly attracted to men. When I look around on the street, I still look at men and not at women, so I think that has remained the same”.*

*(Sarah, 47 years old, woman, pansexual)*

Others reported discovering that their sexual attraction was broader or more flexible than expected, and that they were attracted to people of different gender identities. However, this did not necessarily lead to an adjustment of their self-identification. One partner reported discovering that her sexual attraction was less broad than she had first thought. 

Interestingly, six participants spoke of a sexual attraction to their individual partner. Some explained that they were not generally attracted to people of the same gender identity as their partner. This created a group who changed their self-identification to include their TGD partner and a group who did not change their sexual orientation label. Célia, for example, said she identifies as heterosexual but still is attracted to her TGD partner:

*“I am not bisexual. I have a relationship with my partner. I love my partner and I cannot live without my partner. My partner is everything to me. She is a person I can say anything to without any difficulty. I have a relationship with this person”.*

*(Célia, 51 years old, woman, heterosexual)*

Three interviewed partners reported feeling uncomfortable with how others perceived them in terms of sexual orientation due to their partner’s transitioning. They reported being labelled as “heterosexual” or “lesbian” by others because of the relationship with their partner, despite not identifying as such. Their partner’s transition reduced the visibility of their sexual orientation, which for some participants felt like a loss of a part of their identity, especially when establishing that identity was a difficult process. It was also difficult to observe that others did not always seem to understand that they were in a relationship with their TGD partner but were not sexually attracted to people of their partner’s gender identity. As a result, two of the participants indicated that they occasionally had to justify themselves to others who misidentified them as heterosexual. Lien, among others, indicated that her lesbian identity is an important part of her identity and that its reduced visibility is difficult:

*“I have to defend myself because now everyone expects me to be straight since I am with a man. It’s so hard because I feel like a part of my identity is no longer allowed because I chose to be with Simon”.*

*(Lien, 23 years old, woman, lesbian)*

### 3.2. Dyadic Processes

#### 3.2.1. The Importance of Mutual Commitment

During the interviews, the majority of partners indicated that they considered a gender-affirming transition to be a process of all partners in the relationship. Most participants were therefore strongly involved in their TGD partner’s transition and felt it was also important to be involved. For instance, seven participating partners reported that they tried to attend most of the counselling sessions regarding the transition because they considered it to be a joint process.

Most participants felt that were sufficiently involved in the transition alongside their partner. They identified some aspects that reinforced this feeling. Seven interviewed partners indicated that it was important for them to communicate with their partner about their gender identity and transition. Participants mentioned that it was essential to them that everything related to transitioning could be discussed. In addition, several partners reported that this helped with their own mental process, as this quote from Sarah illustrates:

*“If someone was troubled by something, if certain things were going to change or certain steps were going to be taken, we always had many conversations about that. We really always talked a lot about everything. And if you can tell your partner how you’re feeling, it’s really only half as hard”.*

*(Sarah, 47 years old, woman, pansexual)*

This also highlights the importance of allowing a sufficient amount of time for partners to process the steps taken. Several participants expressed that they were grateful that their TGD partner allowed them time and considered their pace. In addition, six partners mentioned the importance of the TGD partner’s understanding of their individual processes during the interviews. They indicated that it was helpful for them to have their feelings taken into account and their boundaries respected. When this was not the case, participating partners struggled, as in the case of Kim:

*“Sometimes I felt I wasn’t being listened to. I understood that he had certain feelings and that something needed to happen, but I was also caught up in the story and I had feelings about it too, but that was not important. Of course, it’s not nice that I was crying about what he was going to undertake, but that’s how I felt. I felt like my feelings came second”.*

*(Kim, 34 years old, non-binary, lesbian)*

Finally, the evidence suggests that being involved in the transition can also be helpful according to interviewed partners. One example is how some participants helped their partner to choose gender-affirming clothing. Ruth, for example, went shopping with her partner for more gender-affirming clothes when her partner wanted to wear feminine clothes outside the home. Ruth chose clothes for her partner that she felt comfortable with at the time:

*“Then I said to her, “Look, let’s go shopping and see what I think is appropriate for you to wear in the outside world.” That meant going into the men’s collection to look for more feminine pieces. A floral shirt, a first handbag that was still masculine, a first woman’s shoe…”.*

*(Ruth, 56 years old, woman, bisexual)*

Five participants reported that they had made decisions about the transition together with their partner and that they had worked out what felt comfortable for both of them in this process.

#### 3.2.2. Experiences Regarding Intimacy

When asked about intimacy during the interviews, the majority of participants reported that transitioning had little effect on physical intimacy. However, the majority of participants reported an impact on sexual intimacy within their relationship.

The interviews revealed that most participants felt satisfied with the level of physical intimacy. Six participants reported that physical intimacy, such as hugging or kissing, was going well in their relationship and that the gender-affirming transition had little effect on this. Eline, for example, described how she and her partner were still physically intimate despite no longer being sexually intimate:

*“On a physical level, we still walk hand in hand in the streets and when we greet each other, it is with a peck on the lips. So, there is definitely still physical contact. Cuddling on the sofa and all that”.*

*(Eline, 41 years old, woman, not 100% heterosexual)*

For two participating partners, physical intimacy was sometimes a little more difficult. In both cases, this happened mainly when the TGD partner was mentally struggling with their transition. Nevertheless, both partners generally felt comfortable with physical intimacy in the relationship.

Several partners sought and found new ways to be sexually intimate together. Four participants reported that sexual intimacy was different from cisgender partnerships and that they had to be creative in finding solutions. This exploration together required a lot of commitment, according to some interviewed partners, but could also be enjoyable. Kathleen described her experience of this process:

*“It’s trial and error. Everything from square one. You have to find out what he likes, what he doesn’t like, what we like together… But that shows the positive side of a relationship: that you can figure that out together”.*

*(Kathleen, 41 years old, woman, pansexual)*

Each of the four partnerships eventually found ways to meet the needs of both partners, including using sex aids or introducing new sexual partners. 

Four participants reported that sexual intimacy with their partner was a barrier in their relationship. For each of the four participants, this had already been difficult before they initiated the gender-affirming transition and it remained a challenge at the time of the interview. Most of these partners hoped that the medical transition would make their TGD partner feel more comfortable physically and consequently improve sexual intimacy. When this was not the case, some interviewed partners experienced a lack of sexual intimacy, including Emma:

*“It was always difficult, right from the start. I knew that his body was something very difficult, so in the beginning it was mostly one-way traffic, but I was always understanding. Then the surgeries happened and it was better for a while. I felt we had a better connection, because his body was more in line with his true self. But now, in the past year, it is really difficult. There is no one-way traffic anymore. That’s a challenge”.*

*(Emma, 21 years old, woman, pansexual)*

Four other participants felt good about the level of sexual intimacy at the time of the interview. One partner reported having more sex since her partner’s transition and expressed satisfaction with this.

#### 3.2.3. Relational Growth

The interviews show that a transition can be an intense process for a partnership. Several participants concluded that it had affected different aspects of their relationship. For example, five participants said that they and their partner had been through a lot during the transition and now felt stronger in their relationship as a result. Sarah explained in the interview how this strengthened the bond with her partner:

*“It’s mainly made us even closer and more intimate with each other. Because what you share during a transition is so special that it just makes the bond stronger and more special”.*

*(Sarah, 47 years old, woman, pansexual)*

Besides a stronger bond, three participants said that they and their partner had improved their abilities to communicate with each other. During the interviews, it was also mentioned that the gender-affirming transition had made the partners aware of how much they could find support with each other and that a lot of mutual respect grew. Finally, some participants indicated that their TGD partner had become happier during the transition and that this had a positive effect on them and the relationship, as in the case of Lien:

*“I said two years ago, “I don’t want hormones. I don’t want breasts. You can dress like a man and have short hair and wear men’s underwear.” I felt that was far enough. But when I look at where we are now, I am happy with the steps we have taken. It is a struggle for me, but there is also a lot of reward. He really is a completely different person, he is so happy. […] It has also brought a lot of good things and we stand very strong in our relationship”.*

*(Lien, 23 years old, woman, lesbian)*

### 3.3. Perception of Support

#### 3.3.1. Need for Support

The narratives of the participants indicate that partners may experience a need for support when their partner is going through a transitioning process. For example, eight partners reported experiencing a need for professional or informal support in at least one instance during the gender-affirming transition. Lien, for example, expressed a need for psychological support following her partner’s coming out:

*“I struggled with it for a year. My parents didn’t know, so I couldn’t say to my parents, “I want to see a psychologist. Pay for the psychologist.”, because they would want to know why. I couldn’t talk about it, I couldn’t put it into words, so it was difficult”.*

*(Lien, 23 years old, woman, lesbian)*

The extent to which this support was perceived to be necessary varied between interviewed partners. Most reported that they needed substantial support during the transition, while a few reported that they needed little support overall or only at certain times. One participant reported not feeling any need for support. The participants that experienced less or no need for support were all partners who had some knowledge of their partner’s gender identity before the start of the relationship.

The issues with which participants felt they needed support varied widely. Communicating with the TGD partner, changes in sexual intimacy, coping with the partner’s gender identity, coping with changes in the TGD partner and in the relationship, and medical interventions and complications are just a few examples of issues for which participating partners had wanted support. Emma expressed in her interview how difficult her partner’s surgeries were for her. As a result, she expected that she will need psychological support when her partner has a phalloplasty in the future:

*“I know this will be a very difficult time. […] I’ve been to a psychologist previously for a long time and could talk to her about this topic quite well. I think I’ll need to start up therapy again before the phalloplasty. I know myself. I’m not going to wait too long, because I just know that I’m going to need to talk to someone about it”.*

*(Emma, 21 years old, woman, pansexual)*

This evidence suggests that partners may experience a need for support across a range of domains, from their own individual processes to the relational, dyadic processes with their TGD partner. In terms of interactions with their social environment or in public, none of the participating partners reported a need for support. Negative reactions from people in their environment were rather exceptional among the participants in this study. Although some family members or friends were uncomprehending or cut off contact with participants, most reported that this had little impact on them. In addition, the majority of interviewed partners reported that they and their TGD partner experienced few minority stressors such as discrimination and stigma. Seven partners said during their interview that strangers sometimes stare at them, but that this had little impact. Public expression of the TGD partner’s gender identity was not associated with much anxiety in this sample. 

Following their perceived need for support, eight participating partners actively sought informal and/or professional support. Most of the participants sought this support through multiple channels. Four of them sought support from family and friends, three of the partners appealed to their partner for support in difficult moments and seven sought contact with other TGD partners. In terms of professional support, four interviewed partners sought individual psychotherapy and two participants reported seeking relationship therapy.

#### 3.3.2. The Importance of Support

The nine participants in this study indicated that both professional and informal support had been helpful during their process. Receiving support made it easier for several partners to cope with the difficulties and reduced the perceived emotional distress. For Eline, support was very important. She stated that she needed it to be able to continue her relationship in the context of her partner’s transition:

*“I dare not say that I would be sitting here now if I didn’t have the network I had or if I didn’t know how to find a therapist myself”.*

*(Eline, 41 years old, woman, not 100% heterosexual)*

The participants reported several things that were particularly helpful about the support they received. Six participants reported that it was helpful to be given a space to express their experiences. These partners found someone to talk to and/or knew they had someone to vent to, which made the difficulties they were experiencing easier to bear. The following quote from Ruth illustrates this. She was able to go to her therapist with her concerns and found this helpful:

*“The fact that I could talk about it helped. The fact that I could say, “Look, this is hard” or “That’s not hard”. With a lot of other people you cannot share these things. […] With my therapist I had the space to say that it’s not easy”.*

*(Ruth, 56 years old, woman, bisexual)*

In addition, three participants reported that having a supportive social network made a difference to them. They reported that they always had family and friends to turn to. They also felt that the fact that the social environment responded positively to the gender identity of the participants’ partners was helpful. Emma, for example, said during the interview:

*“I do believe that it has made it easier because you know that you are surrounded by all these people who support you. It really makes a big difference”.*

*(Emma, 21 years old, woman, pansexual)*

Furthermore, counselling with a psychotherapist was reported as helpful by six participants. Several participants indicated that the focus in these sessions was on their experiences with the transition and how they dealt with it. Some felt it was important to be able to tell their side of the story and work through it. According to two participating partners, relationship therapy also helped them in the dyadic processes they and their partner were going through. For example, in her interview, Kim explained how relationship therapy helped her and her partner to improve their communication:

*“It helped that there was someone who moderated between us and noticed certain things. We can talk well, but sometimes the actual message of what we’re saying doesn’t come across right away. […] On that level it was helpful. I think we still take the things we learned in therapy with us now. We know that there are things we think about differently and sometimes we have to accept that. What we weren’t so good at before, we are better at now”.*

*(Kim, 34 years old, non-binary, lesbian)*

Finally, seven participants indicated that (online) contact with other TGD partners had been very important and helpful in their process. Several participants expressed the need to find others going through similar events. Sharing experiences, giving advice and supporting each other were cited as reasons why this was such an important source of support. Hearing both positive and challenging experiences from other partners also provided perspective for several participants, including Lien:

*“What I always find most important about the support group is that there is always someone who is in a more difficult situation and there is always someone who has a positive feeling, and that puts things into perspective. You need something to lift you up and you need to know that it’s not all that bad”.*

*(Lien, 23 years old, woman, lesbian)*

#### 3.3.3. Evaluation of Support

Of the eight participants who sought support, three indicated finding the support they needed. The remaining five participants stated during the interviews that they did not find the support they desired. They gave several reasons for this.

Four participants indicated that the support from their social network was not sufficient, although they experienced their supportive environment as helpful. The reason interviewed partners gave for this was that others who were not experiencing their partner’s gender-affirming transition could not fully understand the impact. Kathleen summarised it as follows:

*“If you haven’t been through it, you don’t know what you’re talking about. That’s the big problem. […] A transition is such a unique experience that you actually have to be part of the process to know what it’s like”.*

*(Kathleen, 41 years old, woman, pansexual)*

A general lack of professional support was mentioned by five participants. Several of them reported feeling alone at times when they needed support and that partners were overlooked in the available professional support. The lack of knowledge about TGD issues among health professionals discouraged some participants from seeking professional support. Interviewed partners reported feeling left to their own devices. They mentioned having to seek support themselves and often not knowing where to find it. Kim eventually found the support she needed, but said in the interview that it was not readily available when she needed it:

*“I think the support is there, but you have to go and find it yourself and take the necessary steps yourself. Especially in the beginning. Before we got support, we were at least seven to eight months along, whereas the first eight months is when you really need the help. We had already done a lot of the process ourselves”.*

*(Kim, 34 years old, non-binary, lesbian)*

In terms of professional support, several interviewed partners felt that health care providers sufficiently involved them in communication during consultations about the medical transition. They indicated that the professionals took their experiences and expectations into account. Participants understood that the focus of these consultations was on their partner, but were satisfied with the extent to which their opinions were taken into consideration.

Six participants indicated that they felt there was a lack of contact with other partners. Most of the participating partners sought support through the support group and peer contact organised by the Transgender Infopoint, though online peer support was also mentioned. A large proportion of them reported that this was very helpful, but also indicated that they experienced some shortcomings. For example, many looked for others with similar stories, but some participants reported that they did not find these, for example, because the group present during the meeting was not sufficiently diverse. The frequency and accessibility of the meetings were also mentioned as perceived points of improvement. Lien mentioned these in her interview:

*“I think these evenings help a lot, but they are too infrequent and too far away. If you can’t make it yourself or there aren’t enough people, you have to wait six months. Especially now, because they’re adjusting the way they operate, there hasn’t been a meeting for a long time. I really need one”.*

*(Lien, 23 years old, woman, lesbian)*

## 4. Discussion

This qualitative study examined the experiences and care needs of partners of TGD people in the context of gender-affirming transition. The research questions were (1) “How do partners of TGD people experience their partner’s gender-affirming transition?” and (2) “How do partners of TGD people perceive informal and professional support in the context of their partner’s gender-affirming transition?”. This section discusses how the findings can be interpreted in the context of the research questions and the previous literature, addresses the strengths and limitations of the current study, and offers recommendations for future research.

### 4.1. Experiences of Partners of TGD Individuals

In relation to the first research question regarding partners’ experiences, the findings suggest that a gender-affirming transition typically has an impact on the partner and the intimate relationship. The present findings indicate that the partner and the characteristics of the relationship can experience changes due to transitioning, which is also found in previous research [8,15]. This supports Theron and Collier’s (2013) concept of “co-transition” [15].

Within their intrapersonal processes, the interviewed partners in this study went through a process of acceptance where the TGD partner’s gender identity was reintegrated into the partner relationship. However, the course of this process and the feelings experienced differed between participating partners. On the one hand, a process of gradual, unconscious change in feelings was described, and, on the other hand, a process of conscious, turbulent change in feelings was identified. Ultimately, a progression towards the acceptance of the gender identity and desire to transition was observed in all partners who participated in the current study. It is important to note, however, that this study only included partners who remained in a relationship with their TGD partner during a gender-affirming transition. Following the models of Lev (2004) and Emerson and Rosenfeld (1996) [24,25], the current research confirms that a process of acceptance can be identified and that it can culminate in reconciliation with the gender identity and wish to transition. However, whether this consists of stages and what these stages entail is not as clear as in the models of Lev (2004) and Emerson and Rosenfeld (1996) [24,25]. The experiences of the participating partners in this study are very diverse and cannot be fully captured by these models.

In addition, in the context of the partner’s intrapersonal processes, it is found that a TGD person’s medical transition can bring many concerns. Partners’ perceptions of this have not previously been explored in research, making this a new finding in this study. A medical transition can be an intense process for partners of TGD people, even if they do not experience negative feelings about their partner’s gender identity and transition wish. Partners who participated in the current study reported fears of change in their partner due to medical procedures, as well as fears of medical complications. The findings suggest that gradual changes may make these effects easier to bear. Finally, this study shows that the decision to freeze the gametes of the TGD partner can also be a difficult decision.

It is further found that a gender-affirming transition of a TGD partner can have an impact on a person’s sexual orientation. The finding from previous research that partners question and sometimes redefine their sexual orientation, in terms of self-identification and sexual attraction and in the context of their TGD partner’s transition, is also observed in this study [1,2,8,9,15]. However, the interviewed partners indicate that they do not always experience a change in sexual attraction [1,5,8]. Several participants speak of attraction to their individual partner rather than to a specific gender identity.

In contrast to previous research [8,15,28], few negative feelings are reported in this sample regarding the disclosure of the TGD partner’s gender identity to the social environment and in public. The majority of participating partners reported that the response from family, friends and in public was fairly positive and that the impact was limited. Previous research suggests that coming out to the social environment can be a stressful process [15] and that people experience different minority stressors as well as dyadic stress [8,15,28,29,32,33]. It is possible that partners of TGD people in Belgium experienced fewer negative reactions and minority stressors, but it is more likely that there was a selection bias in the recruitment of the sample. Presumably, mainly partners who experienced personal growth during the transition participated in this study, rather than partners who found transitioning more challenging.

In addition to intrapersonal processes, there seem to be dyadic processes within the partnership. Participants in this study report that a gender-affirming transition is a joint process, rather than a process of the TGD partner alone. The results suggest, in line with previous research [8,15], that this process can have a strong impact on a partnership.

The findings suggest that partners may experience changes in sexual intimacy. As Brown (2010) points out, some partnerships appear to redefine their intimacy during the process of a gender-affirming transition [1]. In the current sample, this could result in the sexual needs of all partners being met. However, not all participants reported finding ways to redefine sexual intimacy. Rather, the evidence suggests that sexual intimacy can remain a barrier for partnerships after transitioning. This contrasts with the findings of previous research, which found that transition can be accompanied by an increase in sexual intimacy [1,8]. The current study also explored physical intimacy, as this has received little attention in previous studies. In contrast to sexual intimacy, physical intimacy seems to change less within a partnership and is less likely to be perceived as a challenge. Consequently, all participating partners in this study reported being satisfied with the level of physical intimacy with their TGD partner.

Mutual involvement between partners emerges as an essential aspect within the dyadic processes. Thus, communication between the partner and the TGD person about the transition process could be very important to coordinate the changes and to help partners in their own mental processes [8]. In addition, the current study identified some elements that may be helpful for partners in coping with changes and integrating gender identity into the relationship. Specifically, interviewed partners indicated that the TGD partner’s understanding of their process, taking small steps within the transition, being given enough time, having input and making decisions together all made a difference. This is consistent with findings from previous research [8].

Finally, this study finds that relationship growth can result from transitioning together. This highlights that the impact of a gender-affirming transition on a partner relationship is not necessarily negative. Several partners in the current study indicate that transitioning has been an intense period in their lives, but that it has resulted in them being stronger in their intimate relationship. In line with previous research, the results of this study suggest that through transition, TGD people and their partner(s) can develop a stronger bond, learn to communicate better with each other and develop greater mutual respect.

### 4.2. Care Needs of Partners of TGD Individuals

In relation to the second research question, the results suggest that partners may experience a need for informal and professional support during the various changes and challenges that can arise during a gender-affirming transition. This finding is consistent with the research of Theron and Collier (2013); however, the current study explores this in more detail [15]. For example, it was found that the extent to which interviewed partners needed support varied. This may be related to when partners are informed of the TGD person’s gender identity. It seems that participants who were informed before the start of the relationship had less need for informal and professional support. Furthermore, the evidence suggests that there is a wide variation in the aspects with which support is desired, ranging from the partner’s individual processes to the dyadic processes within the partner relationship. 

In addition to the need for support, this and previous research have found that informal and professional support can be experienced as helpful by individuals with TGD partners [15]. Receiving support appears to have made it easier for interviewed partners to cope with changes and difficult moments in transition. More specifically, having the space to talk about their experiences was mentioned as important by the participants in this study. A supportive social network was referred to as beneficial to their process, which is also reported in other research [15]. Here, it makes a difference to participating partners to know that they can turn to their social network and that this network is positive about the transition. As in previous studies, this research finds that (online) peer contact can make a difference for partners [15]. A high proportion of interviewed partners appeared to seek contact with people who had experienced similar events. Sharing experiences, exchanging advice and mutual support are mentioned as reasons why this is such an important source of support. Contact with other partners provides perspective and makes participants feel understood. Finally, psychotherapy was also perceived as helpful by partners in this study. 

As the limited available research suggests, partners do not always manage to find the support they are seeking [15]. The evidence indicates that the participating partners experience a general lack of informal and professional support. In terms of informal support, this gap lies primarily in the fact that social network support cannot always meet the needs of the partner. This is a finding that has not emerged in previous research. A second perceived lack of informal support relates to contact with other partners. In fact, partners participating in this study point out that the frequency of peer contact is low and that they do not always find people with similar stories. Within the available professional TGD care, interviewed partners report being sufficiently involved in consultations about their TGD partner’s medical transition. However, current evidence indicates that partners experience a lack of psychological support. Partners in this study report having to seek support themselves, but often do not know where to find adequate professional support. The low level of experience with TGD issues among health care professionals may be a barrier to this.

### 4.3. Clinical Implications

The above findings indicate a need for support among partners of TGD people, albeit to varying degrees and not for all partners. However, even partners who have few difficulties with the gender identity and gender-affirming transition may face some challenges, such as concerns regarding gender-affirming medical interventions. Unfortunately, partners who experience a need for emotional support do not always seem to find it. These findings have several implications for clinical practice.

Firstly, there are some things that therapists themselves can do to ensure that professional counselling is perceived as helpful by partners. For instance, an increased awareness of the partners of TGD people in professional practice is advisable. To achieve this, it is beneficial for health care providers to be aware of the unique experiences of partners of TGD people and the associated complexities. Where appropriate, health professionals should acknowledge their own shortcomings and lack of knowledge on the subject, educate themselves and possibly consult colleagues or refer their client(s). It is also important for them to be aware of their own prejudices towards TGD people. It may be useful to assess the need for support among partners more frequently in consultations regarding the transition of the TGD partner. It may be helpful to refer partners to psychotherapy faster in order to address their reported feelings of being left to their own devices in professional health care.

Secondly, partners of TGD individuals may need a safe environment in which they are heard and in which their feelings and thoughts are given space to exist. In psychotherapy, it is important to acknowledge their feelings in this regard, even if they fluctuate greatly or sometimes seem contradictory. It is, after all, a process. In addition to acknowledging feelings, mental health professionals can help to explore the partner’s identities, whatever they may be and however they may change. Throughout this process, therapists can help partners to come to terms with their sense of loss, but also help them to recognise that their partner is not changing completely, for example, encouraging them to discuss how the transition is taking place, what changes are taking place, what new and old identity traits they recognise in their partner and how they reconcile this with their feelings.

Finally, contact with other partners also emerged as an important source of support for partners. Health care providers can encourage contact with peers as much as possible, and it may be useful to expand professional initiatives for partners to address the perceived shortcomings.

### 4.4. Study Strengths

The current qualitative study has multiple strengths. Although there has been an increase in research on the experiences of TGD people’s partners in recent years, there are still major gaps in the scientific literature on this topic. In addition, there is a relative lack of attention to the specific care needs of this population. The findings of this study therefore contribute to scientific knowledge by addressing the unique experiences and care needs of people that partner with TGD individuals. In addition, this study involves direct questioning of the target population. This provides a valuable insight into the perspectives of the interviewed partners, the care needs they have, and the perceived strengths and limitations of partner care in Belgium. Finally, a heterogeneous sample was recruited in terms of age, relationship duration, sexual orientation and gender identity of the TGD partner. In addition, partners who were aware of their partner’s gender identity before the start of the intimate relationship as well as partners who were informed after the start of the intimate relationship participated in the study. This makes for a diverse sample, covering a wide range of perspectives.

### 4.5. Study Limitations

There are several limitations to this study. Firstly, because of the use of a qualitative research method and convenience sampling, the results have limited statistical generalisability. It cannot be demonstrated that the variability within the recruited sample reflects the variability within the population under study [51]. Indeed, there is a bias towards participants who were willing to share their experiences. It is possible that the partner relationships in this sample are more stable than those in the target population, as research suggests that TGD people in relationships with cisgender women tend to have longer and more stable relationships [52]. Furthermore, with the exception of one participant, the sample consists of female cisgender partners, despite efforts to recruit individuals with other gender identities. Possible explanations are that the social media recruitment call reached mainly female partners or, more likely, that cisgender women are more likely to participate in similar research. Additionally, most participants identified as non-heterosexual. It is unclear whether this is due to the sampling method or a more frequent non-heterosexual identity in partners of TGD individuals. The sample is also homogeneous in terms of the origin of the participants. With one exception, all participants are of Belgian origin. It could be that the recruitment method used did not reach partners with a non-Belgian background, or that partners of non-Belgian origin are less likely to participate in similar studies. Secondly, there is also limited generalisability in terms of qualitative generalisation, in which generalisation is “based upon an attempt to match the variation in the data with the experience and practice op the phenomenon under study” (p. 99) [51]. This is due to the fact that this study did not map the variation within the phenomenon of co-transition. Due to limited resources and in order to keep the study sufficiently focused, only the perspectives of people who remained in a relationship with their TGD partner are discussed. Ex-partners were not included in the current sample. Lastly, despite the efforts made to eliminate researcher subjectivity from the study, this limitation remains.

### 4.6. Suggestions for Future Research

Based on the limitations of this study, some suggestions for future research can be formulated. Future studies could follow TGD partnerships longitudinally. This would help to identify the long-term effects of a gender-affirming transition and identify possible protective and risk factors associated with remaining in an intimate relationship. Another suggestion is to investigate why cisgender women are over-represented in existing studies and how to reach partners with other gender identities. Research on partners with a migration or non-European background could also be valuable. By belonging to two minority groups, and therefore potentially experiencing minority stressors due to more than one status, it is imaginable that these partner experiences will be different. Furthermore, future research could include the ex-partners of TGD people. This could potentially explore why some partners remain in a relationship with their TGD partner and others do not. Furthermore, there is a need for research on intimate relationships where the experiences of all partners in the intimate relationship, including the TGD partners, are considered. Finally, it may be valuable to conduct research among people who entered into a relationship after transitioning had been initiated, as there is still very little research on this.

## 5. Conclusions

The findings from this study add to the existing body of research on the experiences and care needs of partners of TGD people. The evidence shows that a partner’s gender-affirming transition can be a very intense experience, involving a wide range of feelings and changes. For partners, the impact of transitioning appears to be largely at an intrapersonal and dyadic level. More specifically, the results suggest that individuals with a TGD partner, as well as the intimate relationship between them, experience parallel transition processes in which the partner and the partnership can face unique challenges. Despite the obstacles experienced by partners, it appears that relationship growth and partner acceptance of the gender identity and wish to transition are possible. Mutual commitment within the relationship and both informal and professional support are seen by partners as essential in coping with these unique experiences. However, the necessary support is not always found, and there were still some areas of improvement identified regarding the current partner support available in Belgium. The findings show the importance of being mindful of this population within professional health care and of further developing existing support.

## Figures and Tables

**Table 1:** Participant characteristics.

Pseudonym	Age	Nationality	Gender- Identity	Gender- Identity Partner	Sexual Orientation	Relationship Duration	Informed of TGD Identity Partner before Start Relationship
Emma	21	Belgian	woman	trans man	pansexual	4 years	no
Ruth	56	Belgian	woman	woman	bisexual	27 years	no
Sarah	47	Belgian	woman	trans woman	pansexual	7 years	partially
Eline	41	Belgian	woman	woman	not 100% heterosexual	10 years	no
Célia	51	Portuguese	woman	woman	heterosexual	20 years	no
Emilie	31	Belgian	woman	trans man	pansexual	8 years	yes
Lien	23	Belgian	woman	man	lesbian	9 years	no
Kim	34	Belgian	non-binary	non-binary trans man	lesbian	11 years	no
Kathleen	41	Belgian	woman	trans man	pansexual	4 years	yes

## Data Availability

The data presented in this study are not publicly available due to privacy restrictions.

## Appendix A

**Table A1:** Standards for Reporting Qualitative Research (SRQR) ^1^ [43].

No.	Topic	Item	Page Number
	**Title and abstract**		
S1	Title	Concise description of the nature and topic of the study identifying the study as qualitative or indicating the approach (e.g., ethnography, grounded theory) or data collection methods (e.g., interview, focus group) is recommended	1
S2	Abstract	Summary of the key elements of the study using the abstract format of the intended publication; typically includes background, purpose, methods, results and conclusions	1
	**Introduction**		
S3	Problem formulation	Description and significance of the problem/phenomenon studied: review of relevant theory and empirical work; problem statement	1–4
S4	Purpose of research question	Purpose of the study and specific objectives or questions	4
	**Methods**		
S5	Qualitative approach and research paradigm	Qualitative approach (e.g., ethnography, grounded theory, case study, phenomenology, narrative research) and guiding theory if appropriate; identifying the research paradigm (e.g., postpositivist, constructivist/interpretivist) is also recommended; rationale ^2^	4–6
S6	Researcher characteristics and reflexivity	Researcher “characteristics that may influence the research, including personal attributes, qualifications/experience, relationship with participants, assumptions and/or presuppositions; potential or actual interaction between researcher” characteristics and the research questions, approach, methods, results and/or transferability	6–7
S7	Context	Setting/site and salient contextual factors; rationale ^2^	4–6
S8	Sampling strategy	How and why research participants, documents, or events were selected; criteria for deciding when no further sampling was necessary (e.g., sampling saturation); rationale ^2^	4–5
S9	Ethical issues pertaining to human subjects	Documentation of approval by an appropriate ethics review board and participant consent, or explanation for lack thereof; other confidentiality and data security issues	4
S10	Data collection methods	Types of data collected; details of data collection procedures including (as appropriate) start and stop dates of data collection and analysis, iterative process, triangulation of sources/methods, and modification of procedures in response to evolving study findings; rationale ^2^	5
S11	Data collection instruments and technologies	Description of instruments (e.g., interview guides, questionnaires) and devices (e.g., audio recorders) used for data collection; if/how the instruments(s) changed over the course of the study	5
S12	Units of study	Number and relevant characteristics of participants, documents, or events included in the study; level of participation (could be reported in results)	4–6
S13	Data processing	Methods for processing data prior to and during analysis, including transcription, data entry, data management and security, verification of data integrity, data coding, and anonymisation/deidentification of excerpts	5–6
S14	Data analysis	Process by which inferences, themes, etc. were identified and developed, including the researchers involved in data analysis; usually references a specific paradigm or approach; rationale ^2^	5–6
S15	Techniques to enhance trustworthiness	Techniques to enhance trustworthiness and credibility of data analysis (e.g., member checking, audit trail, triangulation); rationale ^2^	6–7
	**Results/findings**		
S16	Syntheses and interpretation	Main findings (e.g., interpretations, inferences, and themes); might include development of a theory or model, or integration with prior research or theory	7–17
S17	Links to empirical data	Evidence (e.g., quotes, field notes, text excerpts, photographs) to substantiate analytic findings	n/a (see “Data availability statement”)
	**Discussion**		
S18	Integration with prior work, implications, transferability and contribution(s) to the field	Short summary of main findings; explanation of how findings and conclusions connect to, support, elaborate on, or challenge conclusions of earlier scholarship; discussion of scope of application/generalizability; identification of unique contributions(s) to scholarship in a discipline or field	18–21
S19	Limitations	Trustworthiness and limitations of findings	21
	**Other**		
S20	Conflicts of interest	Potential sources of influence of perceived influence on study conduct and conclusions; how these were managed	22
S21	Funding	Sources of funding and other support; role of funders in data collection, interpretation and reporting	22

^1^ The authors of the SRQR created the SRQR by searching the literature to identify guidelines, reporting standards, and critical appraisal criteria for qualitative research; reviewing the reference list of retrieved sources; and contacting experts to gain feedback. The SRQR aims to improve the transparency of all aspects of qualitative research by providing clear standards for reporting qualitative research. ^2^ The rationale should briefly discuss the justification for choosing that theory, approach, method, or technique rather than other options available, the assumptions and limitation implicit in those choices, and how those choices influence study conclusions and transferability. As appropriate, the rationale for several items might be discussed together.

## Appendix B

### Interview Guide

1. Can you tell me a little about yourself?
  - Where are you from?
  - How would you describe your ethnicity?
  - How old are you?
  - What is your current relationship status and duration?
  - How do you identify in terms of gender?
  - How do you identify in terms of sexual orientation?
2. Can you tell me a little about your partner?
  - How does your partner identify in terms of gender?
  - In what ways has your partner made gender-affirming changes?
    - Has this had an effect on you?
    - Has this affected your relationship?
    - How did you deal with this?
3. When and how did your partner disclose to you how they felt regarding their gender identity?
  - What was your reaction?
  - How did this make you feel? Did this feeling change over time?
  - Has it affected you and/or your relationship? If so, in what way?
  - How have you dealt with this?
4. Can you tell me a little more about your relationship with your partner?
  - How did you meet your partner?
  - How long have you been together?
  - What are the strengths of your relationship?
  - What are the obstacles in your relationship?
  - What are your experiences regarding emotional, physical and sexual intimacy?
  - How do you view the future of your relationship?
5. How have people around you reacted to your relationship with your partner?
  - Your family
  - Friends
  - School and/or work
  - Strangers, in public
  - How did this make you feel? How did you experience this?
  - How did these different reactions of others affect you and your perception of the relationship?
  - Are these experiences different from experiences in previous relationships?
6. Have you been sufficiently involved in the gender-affirming transition?
  - In what way have you been involved?
  - Were there any particular moments or events that were challenging for you?
  - What were your needs at the time?
  - How did you deal with it?
  - What was and wasn’t helpful to you?
  - What role did support from others (friends, general practitioner, psychologist, etc.) play?
  - What kind of support would have been helpful for you?
  - Is there anything you would have liked to have gone differently?
7. Is there anything you’d still like to add?
